# Supplementary material for: Detection and genetic characterisation of Toxoplasma gondii circulating in free-range chickens, pigs and seropositive pregnant women in Benue state, Nigeria
Source: PLoS Negl Trop Dis. 2021 Jun 2;15(6):e0009458. doi: 10.1371/journal.pntd.0009458 (PMC8202946; doi:10.1371/journal.pntd.0009458)
Supplement: S1 Table — (DOCX) [file pntd.0009458.s001.docx]

**S1 Table. Primer sequences and cycling conditions for host and *Toxoplasma gondii* specific qPCR.**

| **Host** | **Target gene** | **Primer name** | **Primer sequence (5’-3’)** | **Reference** |
| --- | --- | --- | --- | --- |
| Chicken (*Gallus gallus domesticus*) | GAPDH | GAPDH-For1 | CGCAAGGGCTAGGACGG | ^[1]^ |
|  |  | GAPDH-Rev1 | GCGCTCTTGCGGGTACC |  |
|  |  |  |  |  |
| Pig (*Sus scrofa domesticus*) | HPRT | HPRT_F | GGTCAAGCAGCATAATCCAAAG | ^[2]^ |
|  |  | HPRT_R | CAAGGGCATAGCCTACCACAA |  |
|  |  |  |  |  |
| Human  (*Homo sapiens*) | p53 | p53_F2 | TTCCTAGCACTGCCCAACA | ^[3]^ |
|  |  | p53_R1 | CAAATGCCCCAATTGCAGGTA |  |
|  |  |  |  |  |
| *Toxoplasma gondii* | 529bp repeat | Tox_9F | AGGAGAGATATCAGGACTGTAG | ^[4]^ |
|  |  | Tox_11R | GCGTCGTCTCGTCTAGATCG |  |

**References**

^[1]^Blake, D.P., et al., *Eimeria maxima: the influence of host genotype on parasite reproduction as revealed by quantitative real-time PCR.* Int J Parasitol, 2006. **36**(1): p. 97-105.

^[2]^Feng, X., et al., *Selection of reference genes for gene expression studies in porcine skeletal muscle using SYBR green qPCR.* J Biotechnol, 2010. **150**(3): p. 288-93.

^[3]^Wilhelm, J., A. Pingoud, and M. Hahn, *Real-time PCR-based method for the estimation of genome sizes.* Nucleic Acids Res, 2003. **31**(10): p. e56.

^[4]^Hosein, S., et al., *Toxoplasma gondii detection in cattle: A slaughterhouse survey.* Vet Parasitol, 2016. **228**: p. 126-129.
