# Supplementary material for: Detection and genetic characterisation of Toxoplasma gondii circulating in free-range chickens, pigs and seropositive pregnant women in Benue state, Nigeria
Source: PLoS Negl Trop Dis. 2021 Jun 2;15(6):e0009458. doi: 10.1371/journal.pntd.0009458 (PMC8202946; doi:10.1371/journal.pntd.0009458)
Supplement: S2 Table — (DOCX) [file pntd.0009458.s002.docx]

**S2 Table . PCR primers and restriction enzymes used for nested multilocus PCR-RFLP and *in-silico* digest.**

| **Marker** | **Primers (external forward, external reverse, internal forward, internal reverse)** | **Nested PCR (bp)** | **Restriction enzymes,**  **NEB buffers, incubation**  **temperature and time** | **Reference** |
| --- | --- | --- | --- | --- |
| SAG3 | P43S1: CAACTCTCACCATTCCACCC; P43AS1: GCGCGTTGTTAGACAAGACA | 226 | NciI, CutSmart,  37 ºC, 1 h, 2.5% gel | ^[1]^ |
|  | P43S2: TCTTGTCGGGTGTTCACTCA; P43AS2:CACAAGGAGACCGAGAAGGA |  |  |  |
| 5'-SAG2 | 5SAG2-Fext: GCTACCTCGAACAGGAACAC;  5SAG2-Rext: GCATCAACAGTCTTCGTTGC | 242 | MboI, CutSmart,  37 ºC, 1 h, 2.5% gel | ^[2, 3]^ |
|  | 5-SAG2F: GAAATGTTTCAGGTTGCTGC;  5-SAG2R: GCAAGAGCGAACTTGAACAC |  |  |  |
| 3'-SAG2 | 3SAG2Fext: TCTGTTCTCCGAAGTGACTCC;  3SAG2-Rext: TCAAAGCGTGCATTATCGC |  | HhaI, CutSmart,  37 ºC, 1 h, 2.5% gel | ^[3, 4]^ |
|  | 3-SAG2F: ATTCTCATGCCTCCGCTTC;  3-SAG2R: AACGTTTCACGAAGGCACAC |  |  |  |
| BTUB | Btb(ext)F:TCCAAAATGAGAGAAATCGT; Btb(ext)R:AAATTGAAATGACGGAAGAA | 411 | BsiEI + TaqI (double digest),  CutSmart, 60 ºC, 1 h, 2.5% gel | ^[2, 5]^ |
|  | Btb-F: GAGGTCATCTCGGACGAACA;  Btb-R:TTGTAGGAACACCCGGACGC |  |  |  |
| GRA6 | GRA6-F1x:ATTTGTGTTTCCGAGCAGGT;  GRA6-R1:GCACCTTCGCTTGTGGTT | 344 | MseI, CutSmart,  37 ºC, 1 h, 2.5% gel | ^[2, 5]^ |
|  | GRA6-F1:TTTCCGAGCAGGTGACCT;  GRA6-R1x:TCGCCGAAGAGTTGACATAG |  |  |  |
| Apico | Apico-Fext: TGGTTTTAACCCTAGATTGTGG;  ApicoRext: AAACGGAATTAATGAGATTTGAA | 640 | AflII + DdeI (double digest), CutSmart, 37 ºC, 1 h, 2.5% gel | ^[2]^ |
|  | Apico-F:TGCAAATTCTTGAATTCTCAGTT; Apico-R:GGGATTCGAACCCTTGATA |  |  |  |

**References**

^[1]^Prestrud, K.W., et al., *Direct high-resolution genotyping of Toxoplasma gondii in arctic foxes (Vulpes lagopus) in the remote arctic Svalbard archipelago reveals widespread clonal Type II lineage.* Vet Parasitol, 2008. **158**(1-2): p. 121-8.

^[2]^Su, C., X. Zhang, and J.P. Dubey, *Genotyping of Toxoplasma gondii by multilocus PCR-RFLP markers: a high resolution and simple method for identification of parasites.* Int J Parasitol, 2006. **36**(7): p. 841-8.

^[3]^Howe, D.K. and L.D. Sibley, *Toxoplasma gondii comprises three clonal lineages: correlation of parasite genotype with human disease.* J Infect Dis, 1995. **172**(6): p. 1561-6.

^[4]^Grigg, M.E., et al., *Success and virulence in Toxoplasma as the result of sexual recombination between two distinct ancestries.* Science, 2001. **294**(5540): p. 161-5.

^[5]^Khan, A., et al., *Composite genome map and recombination parameters derived from three archetypal lineages of Toxoplasma gondii.* Nucleic Acids Res, 2005. **33**(9): p. 2980-92.
