# Supplementary material for: Detection and genetic characterisation of Toxoplasma gondii circulating in free-range chickens, pigs and seropositive pregnant women in Benue state, Nigeria
Source: PLoS Negl Trop Dis. 2021 Jun 2;15(6):e0009458. doi: 10.1371/journal.pntd.0009458 (PMC8202946; doi:10.1371/journal.pntd.0009458)
Supplement: Attachment — Submitted filename: Reviewers comments.docx [file pntd.0009458.s003.docx]

**Response to Editorial Office.**

We confirm that the maps using in Figure 4 can be published open access. The base layer was access from ARC GIS (version 10.1). These details are now included.

We can confirm that all data has already been submitted and accepted to the relevant public repositories, as stated. Release is delayed until acceptance.

**Reviewer's Responses to Questions**

**Methods**

Reviewer #3: However, authors mention that it was collected sera samples from pregnant women, the T. gondii human serology results were not shown. This result is not essential for the stated objectives, but certainly could improve the understanding of PCR-based methods and sample strategy collection performance to detect parasite DNA in human blood samples.

- This is a good question that has highlighted a gap in our manuscript. The serological survey was published in 2019 (we have now added the reference). Here, we screened blood samples collected from seropositive pregnant women to improve chances for parasite detection and permit comparison of genotypes with those detected in pigs and chickens. We have updated the manuscript to make this clear (lines 169-174 in the revised tracked manuscript and throughout).

**Results**

Reviewer #3: I believe that the inclusion of one more figure describing the sampled LGAs in correlation with the corresponding genotypes isolated from chickens, pigs and pregnant women could contribute to improve the data interpretation concerning the zoonotic character of T. gondii infections.

- We thanks the reviewer for this suggestion. In response, we have revised Figure 3, added text (lines 424-426) and added a new figure (Figure 4).

**Editorial and Data Presentation Modifications**

Reviewer #3: However I suggest some minor edits of existing data which enhance clarity. They are listed below

1- in the title where it reads Prevalence and genetic characterisation of Toxoplasma gondii circulating in free-range chickens, pigs and pregnant women in Benue state, Nigeria I would suggest that the word "prevalence" should not be employed in the title (and also in the manuscript), because the term "prevalence" has an epidemiological connotation, and, what has been done cannot represent a prevalence in epidemiologic terms. I suggest using "occurrence" instead of "prevalence", then it would be: Occurence and genetic characterisation of Toxoplasma gondii circulating in free-range chickens, pigs and pregnant women in Benue state, Nigeria.

- Many thanks for this suggestion. In response, we have amended the title and the rest of the manuscript as suggested. We have changed ‘prevalence’ to ‘detection’ in the title, recognising the use of seropositive women for sampling, not all women.

2-Authors reports that bioassays remain the most sensitive means of detecting Toxoplasma cysts in animal tissues. However it is even more evident that molecular methodologies should be employed for surveys in animal meat samples as demonstrated by Schares and colleagues: International Journal for Parasitology 48 (2018) 751-762, where it is presented a paper entitled: Toxoplasma gondii infections in chickens – performance of various antibody detection techniques in serum and meat juice relative to bioassay and DNA detection methods. In my opinion authors could refer to Schares publication to better value their findings.

- Another good suggestion. In response, we have added reference to the paper by Schares and colleagues in this section (lines 83-84). The work was already quoted elsewhere in our manuscript.

3- Please in the Methods section inform how long, and under what condition (temperature, humidity) Paper Card with human blood samples were kept until processed for DNA extraction. Another important information is: for each patient what was the total quantity of blood in the Paper Card processed for DNA extraction?

- Details added as requested (lines 155-156 and 175). As stated, 125 µl of EDTA-stored blood sample was added to each FTA card. We have now clarified that ~20% of each blood spot was taken for processing.

4- I suggest the inclusion of a new figure describing the samples collected from the LGAs (Ukum, Kastina-Ala, Makurdi, Gboko, Otukpo and Okpokwu for free-range chickens; Ukum, Kastina-Ala, Konshisha, Makurdi, Gboko and Gwer- East for pigs, and Kasitina-Ala, Makurdi and Otukpo for pregnant women) in correlation with the corresponding genotypes	isolated from chickens pig and pregnant women from those LGAs. I believe it could contribute to improve the data interpretation concerning the zoonotic character of T. gondii infections. A figure with the Nigeria map,could spatially gather information from the sampled LGAs with the corresponding genotypes	isolated discriminating from chickens, pigs and pregnant women for each LGA. It is, at certain measures, shown in figure 3 but, it is not so easily understandable in that figure.

- Thanks for this suggestion. In response, we have now added a new figure (Fig. 4).

It is noteworthy that the way the study was conducted, this correlation cannot be straightly established, because one cannot exclude that meat of the animals evaluated in each region, might have been originary from another places and sold in the local market. In addition, patients also cirlulate in different regions during their lives. This is an intrinsic limitation of this type of study and there is no way to avoid it. Nonetheless, this does not invalidate the study presented, however, it should be stated in the manuscript clearly as an intrinsic limitation for this type of study.

- Many thanks for highlighting this limitation. We have now added discussion of this point (lines 546-549).

5-However authors mention that it was collected sera samples from pregnant women, the T. gondii human serology results were not shown. This result is not essential for the stated objectives but certainly could improve the understanding of PCR-based methods and sample strategy collection performance to detect parasite DNA in human blood samples. In this case a new column with serological human information could be added to table 1.

- Many thanks for this question – it has allowed us to identify a gap in our description of the study plan. Specifically, the human samples were selected from a larger sample set described in a previous publication. Here, we prioritised samples from seropositive women to increase the likelihood of detecting *T. gondii* genomic DNA. The manuscript has now been updated to reflect this key point, including the reference, adding text (lines 169-174 and 323-328), and a new table (Table 2 in the revised manuscript).

Also in this, or in a separate table the stage of pregnancy (gestational age of the fetus) if known could be informed. This information can also be valuable for specific education campaigns for the local primary prevention of congenital toxoplasmosis.

- We have added a new table (Table 3) and text (lines 327-328 and 468-469).

6- It is important to be informed if serological evaluation soon after the blood collection (in 2016) and treatment were possible to be provided to pregnant women and their babies. These informations are important to be reported for establishing critic view for the necessity of public health consistent policies to be adopted in all over the world for prevention of this neglected disease.

- Unfortunately, there was a considerable delay between blood collection and serological analysis.

7-Authors conclude (lines 713 -715) that: "free-range chickens and/or pigs shared T. gondii genotypes with pregnant women, representing probable sources of infection to humans in the study area." I´d suggest that authors could consider that instead of writing "representing probable sources of infection to humans" it could be: "representing potential sources of infection to humans", also it should be considered and stated that those genotypes are in environment and that, as well as the animals, humans could be infected with those genotypes from environment, by drinking water and or eating soil and vegetables contaminated.

- Two reasonable points. We have amended the text accordingly (lines 546-549).

**Summary and General Comments**

Reviewer #1: 1.	Line 253—parasitaemia will be appropriate only for blood samples from women but not meat samples from chickens and pigs

- Revised to ‘parasite occurrence’. Please refer to line 38 in the tracked revised manuscript.

2.	Perhaps the authors are unaware of 2 recent reviews for toxoplasmosis in chickens ( Parasitology147, 1263-1289, 2020) and pigs (Vet. Parasit. 288, 109185)

- Many thanks for highlighting these reviews. We have now accessed and cite these relevant reviews as references #2 and #3 (for example line 70).

Reviewer #2: I only have as suggestions:

- A best explanation how PCR contamination was controlled at the lab and what controls were used for this -To include some references that support the findings of PCR positive in blood during prevalence studies and to compare with the finding of the authors:

1.	Parasitol. Res. 101, 619–625..

2.	Acta Tropica 2018; 184:83-87. doi: 10.1016/j.actatropica.2018.01.013.

- Many thanks for these suggestions. We have added text accordingly (lines 182-184 in the tracked revised manuscript) and cited these references in expanded discussion (references #39 and #40, lines 463-46).

Reviewer #3: The present study brings out relevant information on the presence of T. gondii genotypes in Nigeria, where there is practically no information about in this field. The data contextualize its importance for public health (congenital toxoplasmosis). The methodology used to detect genetic material of the parasites is described in the literature and used successfully in the manuscript. Authors present an interesting strategy based on collecting and extracting DNA for forensic research, that is: transfer blood in EDTa to Whatman FTA card for subsequent DNA extraction. This strategy proved to be viable and perhaps could be used as a strategy for locals where the preservation of genetic material might be at risk due to the physical distance and or time between collection and extraction processing.

Thepresented data reinforce the notion that the population structure of T. gondii in Africa, particularly in Nigeria, seems to exhibit lower genetic diversity when compared to the greater diversity observed for south america, and, in the present case, suggesting closest links to archetypal type II T. gondii strains.

- We thank all three reviewers for their supportive comments.
